# pH-Dependent Conformational Changes of KcsA Tetramer and Monomer Probed by Raman Spectroscopy

**DOI:** 10.3390/ijms20112736

**Published:** 2019-06-04

**Authors:** Ann-Kathrin Kniggendorf, David Schmidt, Bernhard Roth, Oliver Plettenburg, Carsten Zeilinger

**Affiliations:** 1 Gottfried Wilhelm Leibniz Universität Hannover, Hannover Centre for Optical Technologies (HOT), Nienburger Straße 17, 30167 Hannover, Germany; bernhard.roth@hot.uni-hannover.de; 2 Gottfried Wilhelm Leibniz Universität Hannover, Naturwissenschaftliche Fakultät, Center of Biomolecular Research (BMWZ), Schneiderberg 38, 30167 Hannover, Germany; david.schmitt@helmholtz-muenchen.de (D.S.); oliver.plettenburg@oci.uni-hannover.de (O.P.); zeilinger@cell.uni-hannover.de (C.Z.); 3 Institute of Medicinal Chemistry, Helmholtz Zentrum München, German Research Center for Environmental Health (GmbH), Ingolstädter Landstrasse 1, 85764 Neuherberg, Germany; 4 Cluster of Excellence PhoenixD, Leibniz University Hannover, Welfengarten 1, 30167 Hannover, Germany

**Keywords:** KcsA, tetramer, monomer, pH-dependent gating, Raman spectroscopy, 532 nm, liposome flux assay

## Abstract

KcsA is a tetrameric potassium channel formed out of four identical monomeric subunits used as a standard model for selective potassium transport and pH-dependent gating. Large conformational changes are reported for tetramer and monomer upon gating, and the response of the monomer being controversial with the two major studies partially contradicting each other. KcsA was analyzed as functional tetramers embedded in liposomes and as monomer subunits with confocal Raman microscopy under physiological conditions for the active and the closed channel state, using 532 nm excitation to avoid introducing conformational changes during the measurement. Channel function was confirmed using liposome flux assay. While the classic fingerprint region below 1800 rel. cm^−1^ in the Raman spectrum of the tetramer was unaffected, the CH-stretching region between 2800 and 3200 rel. cm^−1^ was found to be strongly affected by the conformation. No pH-dependency was observed in the Raman spectra of the monomer subunits, which closely resembled the Raman spectrum of the tetramer in its active conformation, indicating that the open conformation of the monomer and not the closed conformation as postulated may equal the relaxed state of the molecule.

## 1. Introduction

The potassium channel KcsA, originally identified in the bacterium *Streptomyces lividans*, is a standard model for selective potassium transport and gating function [1,2,3]. KcsA is a tetrameric channel formed out of four identical monomer subunits. Each monomer consists of two α-helical transmembrane domains (TM1 and TM2) connected with an extracellular loop leading into a short pore helix and a selective filter structure, responsible for the selectivity of the formed channel to potassium [2,4]. The pore of the channel shows an aqueous cavity with a diameter of 10 Å located below the selectivity filter [2]. The gating process of KcsA is known to be pH sensitive with the channel being open at pH values below pH 5.5 [5,6], and the activation/deactivation cycle itself is subject to hysteresis, with the opening of the channel requiring more energy than its closing [7]. The pH-dependent gating is thought to be caused by a cluster of charged amino acids at the end of TM2 [6,8] and the cytoplasmic domain at the C-terminus [9]. Furthermore, the function of KcsA is inhibited in the presence of external Cs^+^, Rb^2+^, and Ba^2+^-ions, allowing the use of external Cs^+^ ions to confirm the involvement of KcsA in an observed transport process [7,10]. In addition, several dynamic analyses by electron spin resonance (ESR) and electrophysiology in combination with site-directed mutagenesis gave a deep understanding of the KcsA channel and the function of its amino acids and domains [11].

Raman spectroscopy allows to rapidly obtain chemical and structural information about a sample independent of its physical state (gaseous, fluid, or solid). As a purely optical technique, it is reagent- and label-free and, dependent on wavelength and intensity of the excitation, minimally or even non-invasive thus suitable for in vivo analysis [12,13]. Protein conformations and protic functions have been the subject of Raman spectroscopic analyses for many years, including the determination of protein secondary structure and the probing of the dynamics of protein and peptide bond structures [14]. However, because Raman scattering intensity is inversely proportional to the fourth power of the excitation wavelength, most of these works were done with excitation wavelengths in the spectral range of UV or even deep-UV [14,15,16,17], thus, significantly increasing the risk of sample damage or introducing conformational changes of the protein during the analysis [14,18]. However, several works were published detailing successful Raman analyses of proteins and protein conformations performed with visible excitation. For example, Rygula et al. analyzed structure, function, and properties of 26 proteins employing two wavelengths in the visible range (488 and 532 nm) and one in the IR (1064 nm) for excitation [15], while Raman microscopy at 532 nm was used by Čabanová et al. for nanopathology, studying human tissues and body fluids [19], and Sosa Morales and Álvarez characterized the structure of the phosphatidylglycerol (PG) model membranes with this technique [13]. Incidentally, we used confocal Raman microscopy at the same wavelength for the optical, non-invasive detection of structural differences in the human Connexin 26 hemichannel (hCx26) at temperatures above and below its activation temperature of 23 °C. By recording high-precision Raman spectra of purified hCx26 in the liquid phase at 10 °C and 30 °C in detergent with and without Ca^2+^ and lipid (POPC), it was possible to directly identify in experiment the effects of Ca^2+^ and lipids on the conformation of the hemichannel [20].

In this work, we analyzed with confocal Raman spectroscopy at 532 nm the solubilized reconstituted purified tetrameric KcsA channel in its active conformation at pH level 5.5 and at pH 8 when the channel is closed, reporting on clearly visible differences in the spectral range dominated by the CH-stretching modes of methyl- and methylene groups between the open and the closed channel. Furthermore, we studied KcsA monomers under the same parameters (pH 5.5, pH 8), comparing their spectra with the results obtained for the tetramer.

## 2. Results

### 2.1. Liposome Flux Assay—KcsA Channel Function

**Figure 1:**
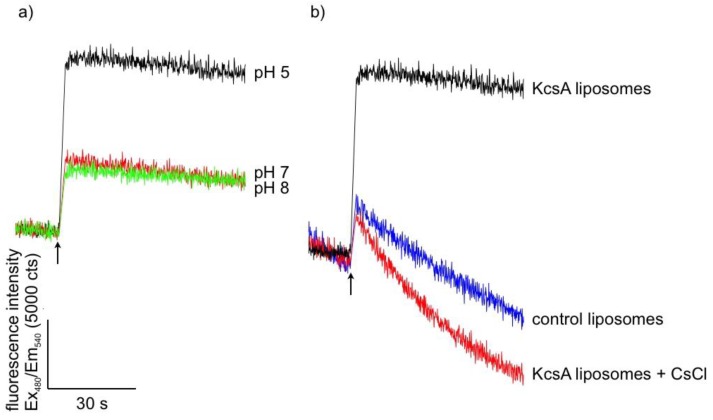
Time-resolved fluorescence signal of purified KcsA reconstituted into asolectin-liposomes at different pH levels (**a**) with pH 5 in black, pH 7 in red, and pH 8 in green, and (**b**) with (red) and without (black) external Cs^+^-ions, and KcsA-free liposomes as control (blue). The moment of KNO_3_ addition is marked with a black arrow, respectively.

Successful reconstitution and channel function of KcsA was confirmed using a liposome flux assay. Liposomes with up to four tetrameric KcsA channels and containing the potassium sensor dye APG-2 were formed as described in Section 4.2. APG-2 containing liposomes without KcsA channels served as control. The transport of K^+^-ions through the KcsA channels was confirmed by recording the increase of the APG-2 fluorescence after addition of KNO_3_ to the respective sample. Figure 1 gives the results for the time-resolved fluorescence of KcsA containing liposomes at different pH levels (a) and in the presence of Cs^+^-ions known to inhibit channel function and a control of KcsA-free liposomes (b). As can be observed by the marked increase in fluorescence intensity in Figure 1a, KcsA rapidly transports injected potassium at pH 5 (black trace), whereas only a very moderate increase occurs at pH 7 (red) and 8 (green) or in the absence of KcsA in the liposomes (blue in Figure 1b). Furthermore, Figure 1b shows markedly lower channel function in the presence of 20 mM CsCl, serving as a source of external Cs^+^-ions known to inhibit potassium transport by KcsA. A small drift is observed in the control liposomes as well as in the KcsA liposomes blocked with CsCl. The reason for this drift is not known.

### 2.2. SDS-PAGE—Confirming KcsA Monomer Samples

**Figure 2:**
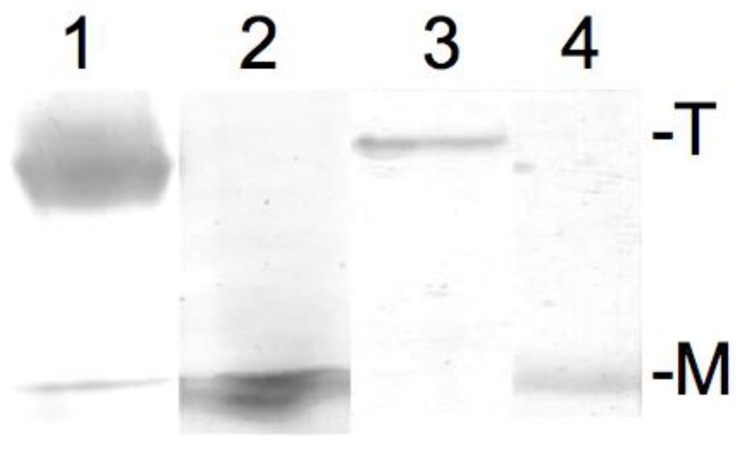
Sodium dodecyl sulfate–polyacrylamide gel electrophoresis (SDS PAGE) of 10 µL of 10 mg/mL KcsA in Dodecylmaltoside detergent at pH 5.5 (tetrameric: lane 1; monomeric after heating: lane 2) and 10 mg/mL KcsA in Dodecylmaltoside detergent at pH 8 (tetrameric: lane 3; monomeric after heating: lane 4). T = tetrameric KcsA, M = monomeric KcsA.

The result of the SDS-PAGE (sodium dodecyl sulfate–polyacrylamide gel electrophoresis) confirming the successful tetramer to monomer dissipation of KcsA in Dodecylmaltoside detergent buffered to pH 5.5 and pH 8, respectively, is given in Figure 2. As can be observed, KcsA monomers were obtained under pH 5.5 and pH 8 (Figure 2, lanes 2 and 4, respectively). A trace amount of monomeric protein band corresponding to approx. one hundred nanograms of protein is visible in the tetrameric fraction (Figure 2, lane 1). However, this was well below the detection limit of the Raman analysis.

### 2.3. Confocal Raman Microscopy—Comparison of the Tetrameric KcsA Channel and KcsA Monomer Spectra

Confocal Raman microscopy was performed on suspensions of liposomes containing the reconstituted tetrameric KcsA channels in a defined state due to the pH level of the sample and on solutions of KcsA monomers buffered to the same pH levels, respectively. As was confirmed by the liposome flux assay (see Section 2.1), the reconstituted KcsA channels are open at pH 5.5 and closed at pH 8.

The Raman spectra held two marked areas of interest, the classical fingerprint region between 300 and 1600 rel. cm^−1^ and the region between 1800 and 3100 rel. cm^−1^, as detailed below. The spectral accuracy of the system was confirmed to be 1 cm^−1^ in both regions.

#### 2.3.1. Fingerprint Region (300–1600 rel. cm^−1^)

**Figure 3:**
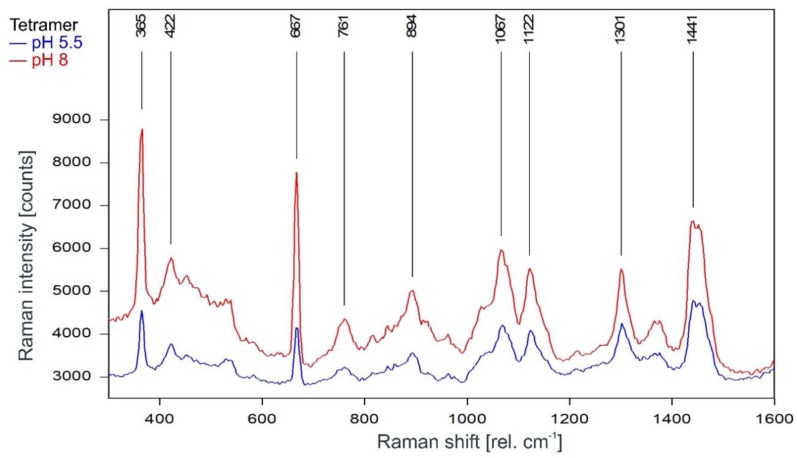
Raman spectrum of the KcsA channel tetramer between 300 and 1600 rel. cm^−1^ in its open state at pH 5.5 (blue) and its closed state at pH 8 (red) as recorded. Raman lines shared by the spectra are marked in black.

**Figure 4:**
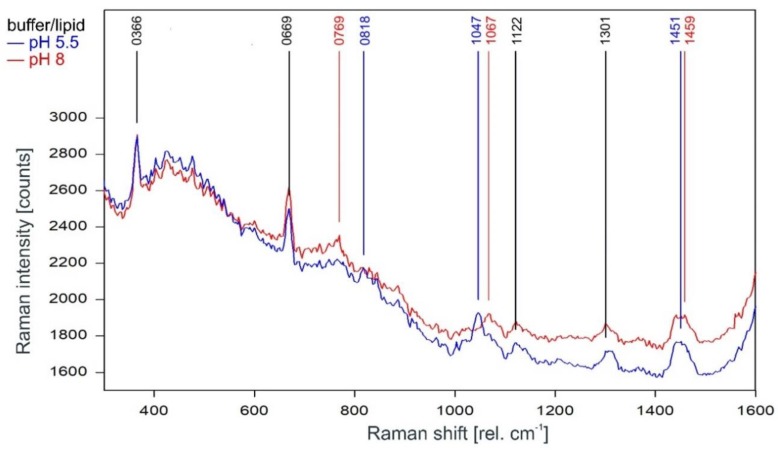
Raman spectrum of the buffer–lipid solution between 300 and 1600 rel. cm^−1^ at pH 5.5 (blue) and pH 8 (red) as recorded. Raman lines shared by the spectra are marked in black.

As can be observed in Figure 3, the Raman spectrum of tetrameric KcsA in the fingerprint region is not affected by the channel state. Except for the Raman line at 894 cm^−1^ the Raman lines of the tetramer spectrum are also found in the spectra of the respective buffer–lipid solutions (Figure 4). However, while a contribution of the buffer–lipid solution to the recorded Raman spectra cannot be ruled out, it is noteworthy that the comparatively strong Raman line at 1047 cm^−1^ in the spectrum of the buffer–lipid solution at pH 5.5 does not appear in the tetramer spectrum, and three of the strongest lines in the tetramer spectrum at 1067, 1122, and 1301 cm^−1^ are weak in the spectra of the buffer–lipid solutions.

**Figure 5:**
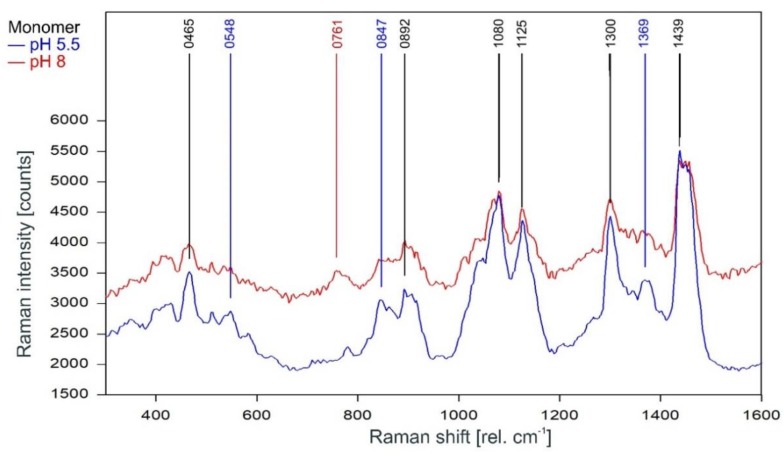
Raman spectrum of KcsA monomer between 300 and 1600 rel. cm−1 at pH 5.5 (blue) and pH 8 (red) as recorded. Raman lines shared by the spectra are marked in black.

Unsurprisingly, the Raman spectra of the KcsA monomers in the fingerprint region are also mostly unaffected by the pH level of the respective sample (Figure 5). There are three marked differences, namely the lines at 548 cm^−1^ and 1369 cm^−1^ in the pH 5.5 monomer spectrum and a line at 761 cm^−1^ in the pH 8 spectrum. However, the additional lines in the pH 5.5 spectrum are also visible in the noisier pH 8 spectrum but do not reach the required signal-to-noise ratio for peak picking. The Raman line at 761 cm^−1^ is also found in the tetramer Raman spectrum and may indicate the formation of a small amount of tetrameric KcsA in the pH 8 monomer sample during the measurement.

**Figure 6:**
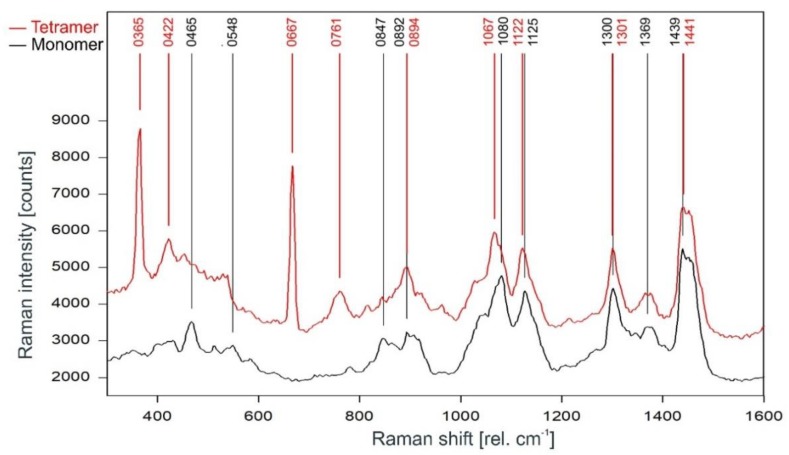
Comparison of the Raman spectrum of the KcsA channel tetramer (red) with the KcsA monomer (black) between 300 and 1600 rel. cm^−1^ as recorded.

A direct comparison of the Raman spectra of the KcsA channel tetramer and the KcsA monomer in the fingerprint region is given in Figure 6. Discounting the Raman lines at 365, 422, and 667 cm^−1^ associated with buffer and lipid (compare Figure 4), most of the lines in the Raman spectrum of the tetrameric KcsA originate from the monomer. However, the Raman line at 761 cm^−1^ in the tetramer spectrum is not seen in the spectra of the monomer and the buffer–lipid solution (see Figure 4 and Figure 5). In addition, a comparatively strong Raman line at 1067 cm^−1^ is followed by a line at 1122 cm^−1^ in the tetramer spectrum, whereas the monomer spectrum holds lines at 1080 and 1125 cm^−1^. From the respective line shapes in the tetrameric and monomeric spectra, one may assume that the lines at 1080 and 1125 in the monomeric spectrum are shifted by 3 cm^−1^ to lower wavenumbers in the tetramer spectrum where they are superimposed by the line at 1067 cm^−1^ not seen in the monomer spectrum. However, the suspected line at 1077 cm^−1^ in the tetramer spectrum does not reach the signal-to-noise ratio (SNR) threshold for peak picking and is only visible as a tiny plateau on the upper flank of the 1067 cm^−1^ line.

The monomer spectrum has additional Raman lines at 465 and 548 cm^−1^ not present in the tetramer spectrum, and a line at 847 cm^−1^ that is significantly stronger in the monomer spectrum than in the tetramer spectrum where its intensity is well below the threshold for peak picking. In addition, there is a slight increase on top of the left edge of the Raman line found at 1441 cm^−1^ in the tetramer spectrum, resulting in this line being detected at 1439 cm^−1^ in the monomer spectrum.

#### 2.3.2. Spectral Region (2800-3100 rel. cm^−1^)

**Figure 7:**
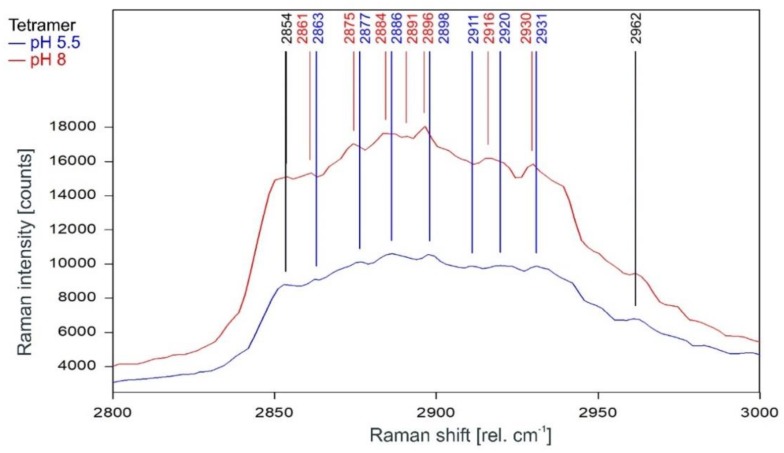
Raman spectrum of the KcsA channel between 2800 and 3000 rel. cm^−1^ in its open state at pH 5.5 (blue) and its closed state at pH 8 (red) as recorded. Raman lines shared by the spectra are marked in black.

**Figure 8:**
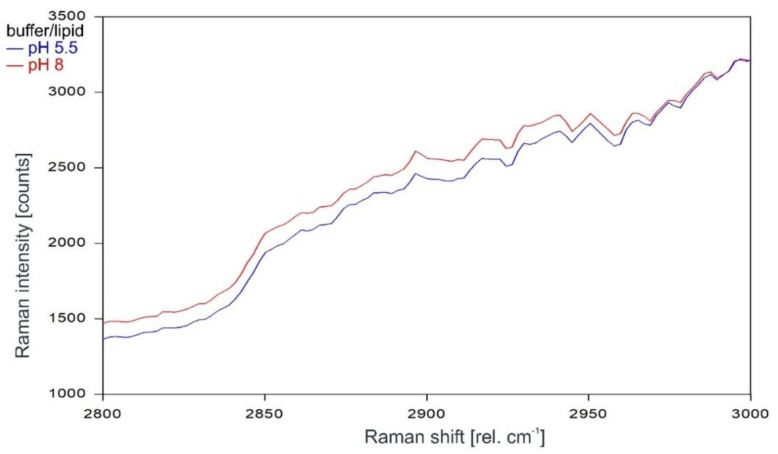
Raman spectrum of the buffer–lipid solution between 2800 and 3000 rel. cm^−1^ at pH 5.5 (blue) and pH 8 (red) as recorded.

Other than in the fingerprint region between 300 and 1600 rel. cm^−1^, the Raman spectrum of the tetrameric KcsA channel between 2800 and 3100 rel. cm^−1^ is strongly affected by the state of the channel. As can be observed in Figure 7, the Raman spectrum of the open channel at pH 5.5 (blue) and the closed channel at pH 8 (red) have only two Raman lines at 2854 and 2962 rel. cm^−1^ in common. Compared to the spectrum of the closed channel, the lines in the Raman spectrum of the open channel are shifted by 2 to 4 cm^−1^ to higher wavenumbers and the line at 2891 rel. cm^−1^ is missing. Figure 8 confirms that the influence of the buffer–lipid solutions on this part of the Raman spectrum is negligible without any Raman lines above the threshold for peak picking and no differential features observed between pH 5.5 and 8.

**Figure 9:**
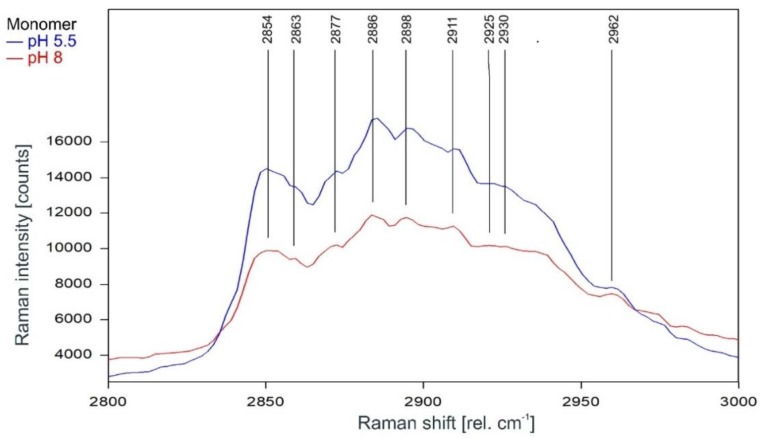
Raman spectrum of KcsA monomer between 2800 and 3000 rel. cm^−1^ at pH 5.5 (blue) and pH 8 (red) as recorded. Raman lines shared by the spectra are marked in black. A comparison of the spectra normalized to the area underneath for clarity is given in Figure 10.

**Figure 10:**
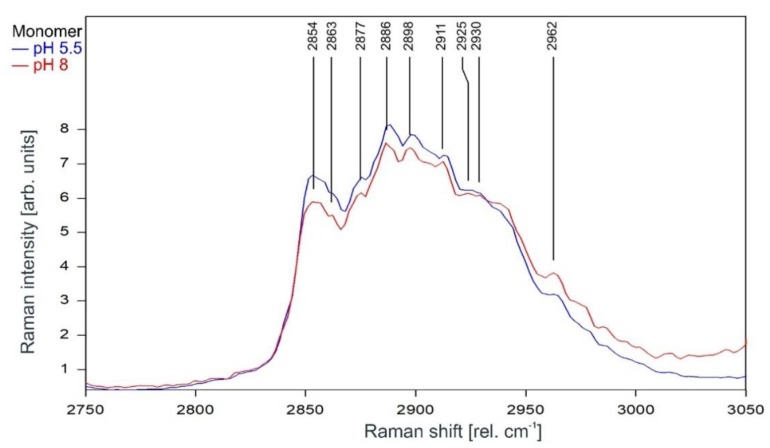
Raman spectrum of KcsA monomer between 2750 and 3050 rel. cm^−1^ at pH 5.5 (blue) and pH 8 (red) normalized to the area underneath the spectra between 2800 and 3000 rel. cm^−1^ for clarity. Raman lines shared by the spectra are marked in black.

As can be observed in Figure 9 and Figure 10, the Raman spectrum of the KcsA monomer is not affected at all by the pH level.

**Figure 11:**
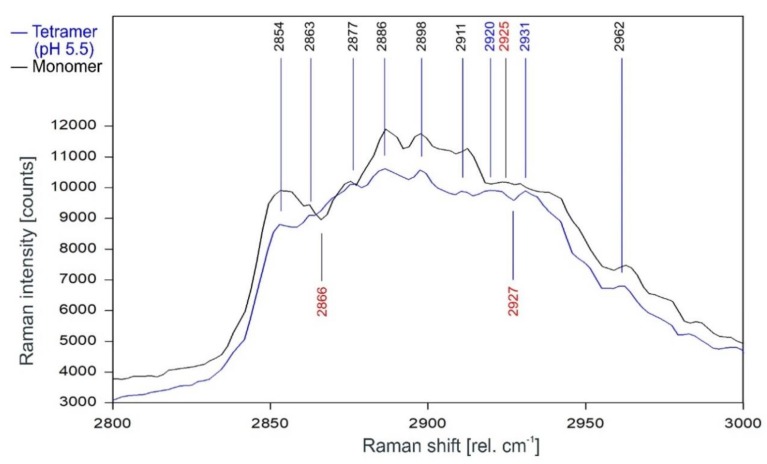
Comparison of the Raman spectrum of the open KcsA channel at pH 5.5 (blue) with the KcsA monomer (black) between 2800 and 3000 rel. cm^−1^ as recorded.

**Figure 12:**
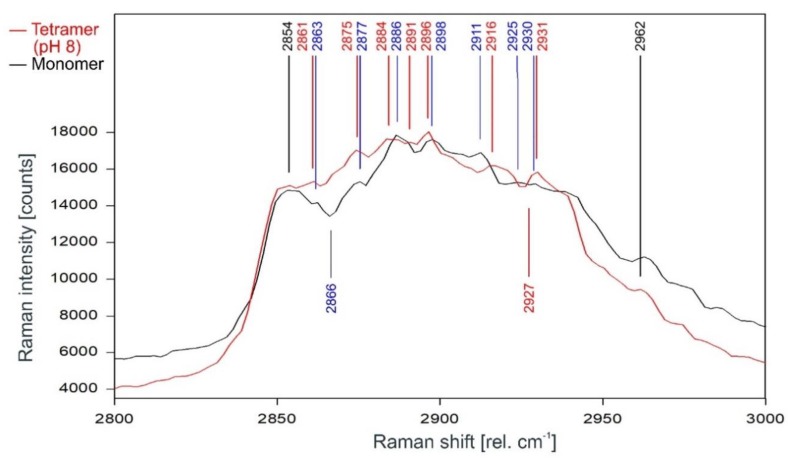
Comparison of the Raman spectrum of the closed KcsA channel tetramer at pH 8 (red) with the KcsA monomer (black) between 2800 and 3000 rel. cm^−1^ as recorded. Raman lines shared by the spectra are marked in black, lines belonging solely to the channel protein are marked in red, exclusive monomer lines are marked in blue.

Figure 11 gives the direct comparison of the Raman spectra of KcsA monomer and the open KcsA channel at pH 5.5. As can be observed, the Raman lines of the KcsA monomer correspond closely with the Raman lines of the KcsA tetramer in its open state. Notable differences occur only in a narrow band between 2915 and 2940 rel. cm^−1^, where the monomer has a Raman line at 2925 rel. cm^−1^ while the open tetramer shows lines at 2920 and 2931 separated by a dip at 2927 rel. cm^−1^, and a pronounced dip at 2866 rel. cm^−1^ in the monomer spectrum not found in the tetrameric spectra (see also Figure 12).

The direct comparison of the Raman spectra of KcsA monomer with the KcsA tetramer in its closed position at pH 8 is given in Figure 12. As can be observed, the differences between the monomer and the closed tetramer are very similar to the differences observed between the spectra of the opened and the closed tetramer, with the noted exceptions of the dip at 2866 rel. cm^−1^ in the monomer spectrum which does not appear in either tetramer spectrum and the dip at 2927 rel. cm^−1^ in the tetramer spectrum missing in the monomer spectrum.

## 3. Discussion

The liposome flux assay reported in Section 2.1 confirmed that KcsA is indeed responsible for rapid potassium transport activity within a second after the injection of potassium into the liposome suspension and that the KcsA channel is predominantly active at pH 5.5 and inactive at pH 7 and 8, as described earlier [5,6]. Furthermore, the liposome flux assay confirmed also that appropriately pH-buffered liposome suspension provides KcsA channels predominantly in the desired state for Raman analysis.

The subsequent Raman analysis of the KcsA channel in its open and closed state revealed, in comparison with the respective monomers analyzed in the same medium and pH conditions, that the Raman spectrum of the tetrameric KcsA channel is independent of the channel state in the fingerprint region between 300 and 1800 rel. cm^−1^ (Figure 3). However, the channel state has a notable influence on the spectrum in the spectral region between 2800 and 3000 rel. cm^−1^ associated with the C–H stretching modes [21]. Most of the Raman lines in this region are shifted by 2 cm^−1^ to lower wavenumbers, and additional lines appeared at 2891 and 2916 rel. cm^−1^ in the closed state while the lines at 2911 and 2925 rel. cm^−1^ disappeared. However, the Raman lines at 2854 and 2962 rel. cm^−1^ framing the shifted lines are unaffected (Figure 7). Moreover, in this region, the spectrum of the open channel closely matches the spectrum of the KcsA monomer, which is not affected by the pH conditions at all (Figure 11). This observation agrees with the conformational model for KcsA gating proposed by Hirano et al. [22], who described that the four cytoplasmic domains of the KcsA monomers form an oligomer in the closed state (pH 7), which is destabilized by positively charged amino acids in the open state [22]. Since the conformational change of the monomer is thought to occur because the equal charges of the monomer subunits in the tetramer repel each other [8], it makes sense that the conformation of the isolated monomers does not change in the absence of a repelling charge, as evidenced by the Raman spectrum of the monomers showing no pH-dependence at all. Since the Raman spectra of the tetramer in its opened and closed state differ markedly (see Figure 7) and large conformational changes of the monomers are proposed in the literature [8,23,24,25], we exclude the option of a pH-induced conformational change of the monomer not being detected.

**Figure 13:**
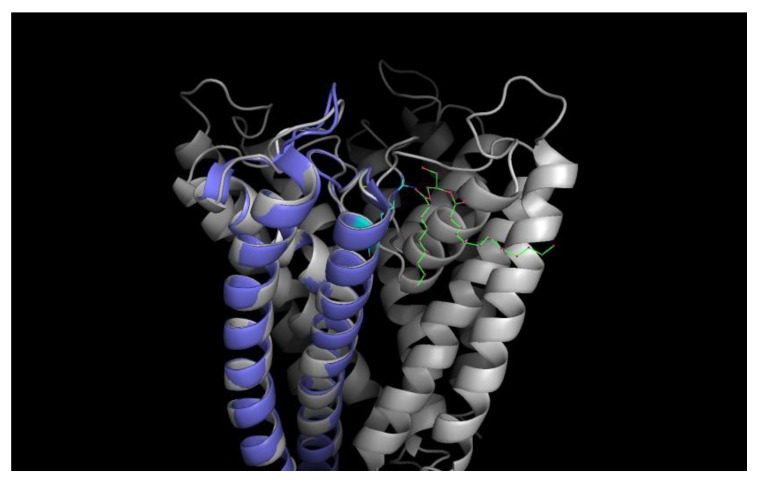
Cartoon of the tetrameric KcsA channel with a negatively charged non-annular lipid (diaglycerol; green-red) given in colored stick representation between the monomeric subunits electrostatically bound to the Argenine residue at position 89 (cyan–blue); one subunit colored violet for clarity. (Structural data obtained from the pdb database (PDB ID: 1k4c); edited with PyMol 1.6.0.0; Build Copyright by Schrodinger, LCC).

However, the pH-independence of the monomer differs markedly from the results of Chill et al., who probed the conformation of KcsA monomers embedded in sodium dodecyl sulfate (SDS) micelles by NMR spectroscopy and hinted at an inherent pH-dependency of the monomer conformation [23]. However, Chill et al. worked with monomers with truncated cytoplasmic domains embedded in SDS micelles, noting a significant effect of the charged SDS environment on the also charged amino acid side chains thought to be involved in the gating mechanism [8,23]. Therefore, it is possible that the monomer conformational change observed by Chill et al. was caused by the positive charge of the SDS micelles repelling the also positively charged cytoplasmic domains of the embedded monomers at pH 4. Since Valiyaveetil et al. [26] demonstrated that lipids, such as PG and POPG, are required for the stabilization and function of the KcsA tetramer, noting that the presence of anionic lipids is a requirement for the gating of the KcsA tetramer (see Figure 13 for a schematic), and results by Triano et al. [27] indicate that SDS may bind specifically to the non-annular lipid binding positions of the monomer, it is likely that the SDS micelles used by Chill et al. cause a similar monomer conformation as if the monomer were part of a tetramer with non-annular lipids present. However, while we confirmed the activity of the tetrameric KcsA channel at pH 5.5 (see Figure 1), it cannot be ruled out that the conformation of the isolated KcsA monomers does change at a pH value lower than that of the tetramer in liposomes.

It is noteworthy that the Raman spectrum of the isolated monomers equals the spectrum of the open channel rather than the spectrum of the closed channel (compare Figure 11 and Figure 12), indicating that—other than postulated by Hirano et al. [8]—the conformation of the monomer subunit in the open state of the tetramer equals the relaxed state of the monomer molecule, whereas the conformation of the monomer subunit in the closed state is the result of a force acting upon it. Interestingly, the binding sites of the lipids required for tetramer stability and function have also been found to prefer anionic over zwitterionic phospholipids [28], further strengthening the assumption that an anionic charge is required to pull the subunits together in the closed state of the tetramer, whereas increased positive charges of the monomers at acidic pH neutralize its effect and allow the monomers to return to their relaxed conformation, thus open the tetramer.

## 4. Materials and Methods

### 4.1. Chemicals, Bacterial Strain Protein Synthesis, and Purification

POPC (1-palmitoyl-2-oleoyl-sn-glycero-3-phosphocholine) and POPG (1-hexadecanoyl-2-(9Z-octadecenoyl)-sn-glycerol-3-phospho-(1′-rac-glycerol)) were obtained from Avanti Polar Lipids Inc. (Alabaster, Alabama, USA). Asolectin was purchased from Sigma-Aldrich Chemie GmbH (Munich, Germany), 6% Cobalt-IDA Agarose from Jena Bioscience GmbH (Jena, Germany), OG (n-octyl-β-D-glucopyranoside) from Carl Roth GmbH & Co. KG (Karlsruhe, Germany), and Asante Potassium Green-2 TMA+ salt (Asante Potassium Green-2 (APG-2)) from Abcam plc (Cambridge, UK).

The *Escherichia coli* strain BL21(DE3) (Life Technologies GmbH, Darmstadt, Germany) was used to host the plasmid pTrcHis2Topo containing the KcsA gene. *E. coli* cells were cultivated in Luria Bertani medium (LB) using ampicillin as a selection marker (100 µg/mL) at 37 °C for 6 h. Synthesis and purification of recombinant KcsA were performed as described earlier [29]. In brief, *E. coli* cells were lysed using a French press and solubilized in 1% N-lauroylsarcosin (NLS). The His-tagged protein was purified by affinity chromatography using a 6% Co-IDA Agarose resin. During purification, the detergent was changed to 30 mM OG.

The concentration of the protein was calculated based on the estimated absorption at 280 nm with an extinction coefficient of 36,440 M^−1^ cm^−1^ and adjusted to 1 mg/mL. The purified KcsA protein was subsequently frozen in liquid nitrogen and stored at −80 °C.

### 4.2. Reconstitution and Testing of Channel Activity

For one batch (1 mL liposomes), 10 mg of asolectin (from soybeans) was weighed in a glass flask (pointed flask) and dissolved in 200 µL chloroform. By means of a nitrogen stream, the chloroform was evaporated while the flask was rotated so that a lipid film formed on the glass surface. The lipid film was dissolved in a pH 7 buffer containing 10 mM Tris (2-Amino-2-(hydroxymethyl)propane-1,3-diol), 100 mM glycine, and the dye APG-2 in a concentration of 5 µM. All steps following the application of the dye were carried out in darkness, and the suspension was stored on ice. After the lipid film was resuspended, the suspension was sonicated to ensure the formation of unilamellar liposomes. One hundred micrograms of channel protein were added to 1 mL of the liposome suspension and incubated for twelve hours at 4 °C. A PD-10 desalting column from GE Healthcare (Chalfont St. Giles, Buckinghamshire, UK) was used to remove the free dye. A fraction of approximately 1 mL of liposomes was collected.

Before testing the channel function, the reconstitution efficiency was analyzed using Cy5 labeled KcsA with a calculated Cy5/KcsA(-tetramer)-ratio of 8.

The ratio of KcsA channels per liposome was calculated based on approximated 2∙10^14^ liposomes per ml (calculated with the assumption that all liposomes are unilamellar with a diameter of about 75 nm) [30]. The Cy5-KcsA concentration in the sample was measured at a wavelength of 650 nm using a nanodrop spectrophotometer. Based on the measured Cy5-KcsA concentration, an average ratio of three to four KcsA channels per liposome was calculated.

A volume of 100 µL liposome suspension was used for the subsequent measurements.

The measurements were carried out in a black 96-well microtiter plate using a Mithras LB 940 plate reader (Berthold Technologies GmbH & Co. KG, Bad Wildbad, Germany). Liposomes were pipetted to 100 µL (final volume 200 µL) of either a pH 5.5 buffer containing 100 mM MES (2-(N-morpholino)ethanesulfonic acid) or to a buffer containing 10 mM Tris and 100 mM glycine (adjusted to pH 7 and pH 8).

During the measurement, 50 µL of a 1 M KNO_3_ solution was added to the samples with an injector after reading the base-fluorescence for 15 s. The subsequent change in fluorescence was measured for 30 s after K-injection. For excitation, a wavelength of 488 nm was used, and emission was measured at the wavelength of 540 nm at a sample rate of 0.1 s.

**Figure 14:**
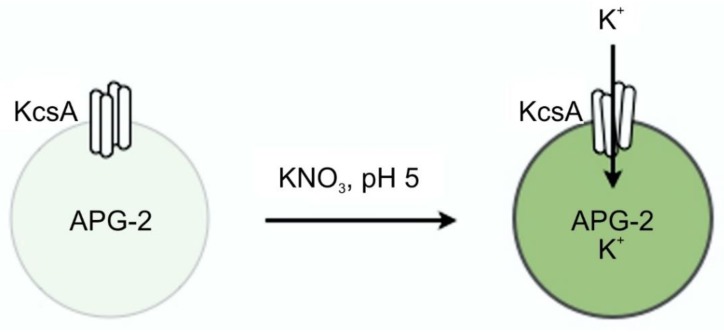
Principle of the liposome flux assay. Potassium ions transported through the KcsA channel open at pH 5.5 cause the dye APG-2 enclosed in the liposome to fluoresce at 540 nm upon excitation with 488 nm.

A schematic illustrating the liposome flux assay technique is given in Figure 14.

### 4.3. Preparation of KcsA Monomers

For dissipating KcsA channel tetramers into KcsA monomers and to hinder the formation of aggregates in the Dodecylmaltoside detergent, the heating protocol given in Table 1 was developed and used in a thermo cycler. The step by step heating process and equilibration of the tetramer to monomer transition ensured a stable solubilization of the monomers. However, direct heating at temperatures of 80 °C and more caused indissoluble precipitates in Dodecylmaltoside detergent.

For the subsequent analysis, the KcsA monomers were diluted into detergent buffered to pH 5.5 (100 mM MES-NaOH, pH 5.5, 10 mM Dodecylmatoside) and detergent buffered to pH 8 (100 mM Tris-HCL, pH 8, 100 mM NaCl, 10 mM Dodecylmatoside), respectively. The samples were filtrated with an 0.45 µm filter to remove possible precipitates and concentrated to 1 mL using a YM30 centrifugal filter with a cut-off of 10 kDa, diluted 10-fold with the respective buffer, and concentrated again to a final protein concentration of 6.5 mg/mL. Before the subsequent Raman analysis, an SDS-PAGE (sodium dodecyl sulfate–polyacrylamide gel electrophoresis) was done with 10 µL of the respective sample solutions for validation.

### 4.4. Raman Spectroscopy

Raman spectroscopy was performed with a confocal Raman microscope (CRM200 by WITec GmbH, Ulm, Germany), equipped with a standard objective (Nikon CFI LU Plan, 50×, NA 0.55, Nikon Instruments Europe BV, Amsterdam, The Netherlands). A frequency-stabilized, frequency-doubled continuous-wave Nd:YAG laser at 531.9 nm was used for excitation. The slit width was 50 µm, realized by a multimode fiber connecting the Raman microscope and the spectrometer (UHTS 300 by WITec GmbH, Ulm, Germany). The laser intensity was set to 36 mW. The loss within the optics prior sample contact was 30%. Raman spectra were recorded with an electron multiplying charge-coupled device (emCCD) camera (Andor DU970N-BV-355, Andor Technology Ltd, Belfast, Northern Ireland), electrically cooled to −69 °C.

For obtaining a first overview of the spectral features of the KcsA spectrum, the system was initially operated with a 600 l/mm grating in the spectrometer. The overview spectra recorded with this setup covered the range between −120 and 3500 rel. cm^−1^ with a spectral resolution of 5 cm^−1^. Here, integration time per spectrum was 100 s, limited by detector saturation in the strong H_2_O Raman feature between 3000 and 3500 rel. cm^−1^.

High-precision Raman spectroscopic measurements were performed subsequently, using a grating with 1800 L/mm. The spectral resolution of the system in this configuration was 1 cm^−1^ with a spectral accuracy of 1 cm^−1^. The integration time per spectrum in this configuration was 20 s, resulting in an average of 50,000 counts for each of the dominant Raman lines in the spectrum.

The Raman measurements were performed with purified KcsA in 30 mM octylglucoside and 1.2 mM POPC/POPG (3:1) using the following buffers: 10 mM Tris, 100 mM NaCl, adjusted to pH 8 and 10 mM MES, 100 mM NaCl, adjusted to pH 5.5. Protein concentration in the measured samples was adjusted to 10 mg/mL in a volume of 200 µL.

For Raman measurements, the sample was mounted on an uncoated 1.2 mm deep indentation slide with a 0.17 mm coverslip sealed with acrylic lacquer to avoid evaporation during the measurement.

### 4.5. Data Extraction and Analysis

Raman spectra used for analysis were averaged over 50 individual spectra using WITec ScanCTRL software (WITec GmbH, Ulm, Germany) to minimize statistical noise components. The analysis of the averaged Raman spectra was done with commercial spectral analysis software (OPUS 5.5 by Bruker Optik GmbH, Ettlingen, Germany) as previously described in [31].

Briefly, the dark spectrum with an intensity of approximately 800 CCD counts per spectrum was subtracted from all data sets. The analysis of the Raman data was done in two steps: peak analysis for each spectrum, and direct comparison of the respective spectra. The peak analysis consisted of determining the position and intensity of the peak (automated peak picking for maxima in OPUS) for each Raman line above the noise. Comparisons of two spectra were made directly based on peak position and line shapes. Only peaks exceeding the average height and width of the noise jitter by at least a factor of two were considered in the final evaluation.

## 5. Conclusions

The Raman spectrum of the KcsA tetramer between 2800 and 3100 wavenumbers is indicative of the pH-dependent channel state with the spectrum of the open state being similar to the spectrum of the KcsA monomer. Since the Raman spectrum of the KcsA monomer is not affected by the pH level, this may indicate that the conformation of the monomer subunits in the open conformation of the tetramer instead of in the closed formation equals the relaxed state of the monomer.

## Figures and Tables

**Table 1:** Heating scheme for KcsA monomer formation

Temperature [°C]	Duration [min]
30	10
35	5
40	5
45	5
55	5
65	10
70	30
80	10

## Abbreviations

APG-2	Asante Potassium Green-2 TMA+ salt
emCCD	Electron multiplying Charge Coupled Device
ESR	Electron Spin Resonance
IR	Infrared
MES	2-(N-morpholino)ethanesulfonic acid
NLS	N-lauroylsarcosin
NMR	Nuclear Magnetic Resonance
OG	n-octyl-β-D-glucopyranoside
PG	Phosphatidylglycerol
POPC	1-palmitoyl-2-oleoyl-sn-glycero-3-phosphocholine
POPG	1-hexadecanoyl-2-(9Z-octadecenoyl)-sn-glycerol-3-phospho-(1′-rac-glycerol)
SDS	Sodium dodecyl sulfate
SDS PAGE	Sodium dodecyl sulfate polyacrylamide gel electrophoresis
TM	Transmembrane domain
Tris	2-Amino-2-(hydroxymethyl)propane-1,3-diol
UV	Ultra violet
VIS	Visible light
